# Recent Advances in Regenerative Therapies in Periodontology

**DOI:** 10.3390/dj13120564

**Published:** 2025-12-01

**Authors:** Andrei-Mario Bădărău-Șuster, Edwin Sever Bechir, Zsuzsanna Bardocz-Veres, Ana Petra Lazăr, Alexandru Vlasa, Mircea Suciu, Tatiana-Maria Coman, Luminița Lazăr

**Affiliations:** 1 Doctoral School of Medicine and Pharmacy, George Emil Palade University of Medicine, Pharmacy, Science and Technology of Targu Mures, 38 Gh. Marinescu Str., 540142 Targu Mures, Romania; 2 Department of Oral Rehabilitation and Occlusology, Faculty of Dental Medicine, George Emil Palade University of Medicine, Pharmacy, Science and Technology of Targu Mures, 38 Gh. Marinescu Str., 540142 Targu Mures, Romania; 3 Department of Periodontology, Faculty of Dental Medicine, George Emil Palade University of Medicine, Pharmacy, Science, and Technology of Targu Mures, 38 Gh. Marinescu Str., 540142 Targu Mures, Romania; alexandru.vlasa@umfst.ro (A.V.); luminita.lazar@umfst.ro (L.L.); 4 Department of Orthodontics, Faculty of Dental Medicine, George Emil Palade University Medicine, Pharmacy, Science, and Technology of Targu Mures, 38 Gh. Marinescu Str., 540142 Targu Mures, Romania

**Keywords:** periodontal regeneration, guided tissue regeneration, guided bone regeneration, biomaterials, platelet concentrates, enamel matrix derivatives, hyaluronic acid, stem cells, laser therapy

## Abstract

**Background/Objectives**: Periodontal regeneration remains a primary goal in contemporary periodontal therapy, aiming to restore both the structural and functional integrity of tissues lost due to periodontitis. Recent advancements in biomaterials, growth factors, and biologically active matrices have expanded the therapeutic possibilities in clinical practice. This narrative review aimed to summarize recent developments in regenerative approaches in periodontology, emphasizing their biological principles, clinical outcomes, and current limitations. **Methods**: A literature search was conducted in PubMed Central and Scopus for randomized controlled trials and clinical trials published between January 2015 and July 2025. Human studies in English, available in open access and evaluating periodontal regenerative approaches, were included, while animal, in vitro, and non-clinical studies were excluded. A total of 67 articles met the eligibility criteria. Data were synthesized in both tabular and narrative form. **Results**: Most trials reported clinically relevant improvements in probing depth reduction, clinical attachment gain, and defect fill when regenerative biomaterials were applied in appropriately selected intrabony defects, although outcomes varied according to defect morphology, surgical protocol, and patient-related factors. **Conclusions:** Although substantial progress has been made, true periodontal regeneration remains challenging. Regenerative techniques such as GTR/GBR, EMD, platelet concentrates, and hyaluronic acid show favorable outcomes in appropriately selected cases, although overall predictability remains limited by variability in study design and short follow-up periods. High-quality, standardized RCTs are needed to consolidate current evidence and support guideline-based clinical decision-making.

## 1. Introduction

Periodontitis is a chronic inflammatory disease characterized by dysbiosis in the oral cavity. Although this disease is being intensively studied, its complete etiology remains unknown. It develops based on untreated gingivitis, with the inflammation progressing to the tooth-support tissue [1]. More severe forms of periodontitis have been observed in patients with systemic conditions, such as rheumatoid arthritis [2], diabetes mellitus [3], and cardiovascular diseases [4,5]. Also, not only do systemic diseases affect the periodontal tissues, but periodontal inflammation also worsens general diseases, the relationship between them being bidirectional [6,7,8]. Recent research emphasizes the fact that periodontal disease has been and will remain a public health problem, with an increase in the prevalence of this condition being reported globally [9,10]. Thus, prompt diagnosis and treatment are very important to reduce the consequences of this condition.

Periodontal treatment aims to eliminate the etiological factor and restore local homeostasis. Persistent periodontal inflammation leads to bone loss in both vertical and horizontal planes. To stop the progression of periodontal disease, the European Federation of Periodontology (EFP) has developed standardized guidelines to help clinicians provide optimal treatment for their patients [11,12]. After behavioral changes, subgingival instrumentation, patients eligible for surgical therapy are those who have a periodontal probing depth greater than 5 mm with bleeding upon probing following the second step of periodontal therapy [13]. Open Flap Debridement (OFD) is a periodontal surgical procedure designed to provide access to root surfaces and alveolar bone for comprehensive debridement and reduction in periodontal pockets, thus promoting tissue healing and long-term periodontal stability. Periodontal regenerative therapies have shown more promising results compared to OFD alone [14]. More and more treatments aim to achieve periodontal regeneration. Periodontal repair involves restoring function without completely rebuilding the original structure (usually through the appearance of a long junctional epithelium), while periodontal regeneration, in addition to restoring function, involves the complete reconstruction of all lost components (root cementum, periodontal ligament, alveolar bone). From a histological perspective, true periodontal regeneration is defined by the reappearance of Sharpey’s fibers, which anchor within newly formed cementum and alveolar bone. This intricate fiber insertion is essential for restoring the functional integrity of the periodontal ligament and re-establishing a stable connection between the tooth and its supporting structures [15]. Thus, periodontal regeneration is the ultimate goal, but it is much more difficult to achieve clinically and requires biomaterials, growth factors, or cell therapies [16].

Advances in biomaterials, biologically active agents, and minimally invasive surgical techniques have expanded the possibilities for periodontal regeneration. However, despite these developments, the current literature remains heterogeneous, with variable clinical outcomes and a lack of consensus regarding the most effective regenerative approaches. These gaps in evidence highlight the need for a comprehensive synthesis of recent data to clarify the current status and future perspectives of regenerative periodontal therapy.

This paper aimed to review and synthesize the most recent developments in regenerative approaches within periodontology, highlighting their clinical relevance and potential future directions.

## 2. Materials and Methods

### 2.1. Eligibility Criteria

Inclusion criteria:

- Scientific articles published from 1 January 2015 to 10 July 2025;
- Randomized Controlled Trials or Clinical Trials that are published in English;
- Open Access Articles;
- Articles regarding regenerative approaches used in periodontology.

Exclusion criteria:

- Animal studies;
- In vitro studies;
- Studies lacking full-text accessibility or comprehensive methodological data;
- Manuscripts with no clear report of a clinical study.

### 2.2. Literature Search Strategy

Although this study does not represent a systematic review, the literature selection and reporting process were structured according to the transparency principles outlined in the Preferred Reporting Items for Systematic Reviews and Meta-Analyses (PRISMA) 2020 guidelines, ensuring methodological rigor and reproducibility [17].

**Figure 1:**
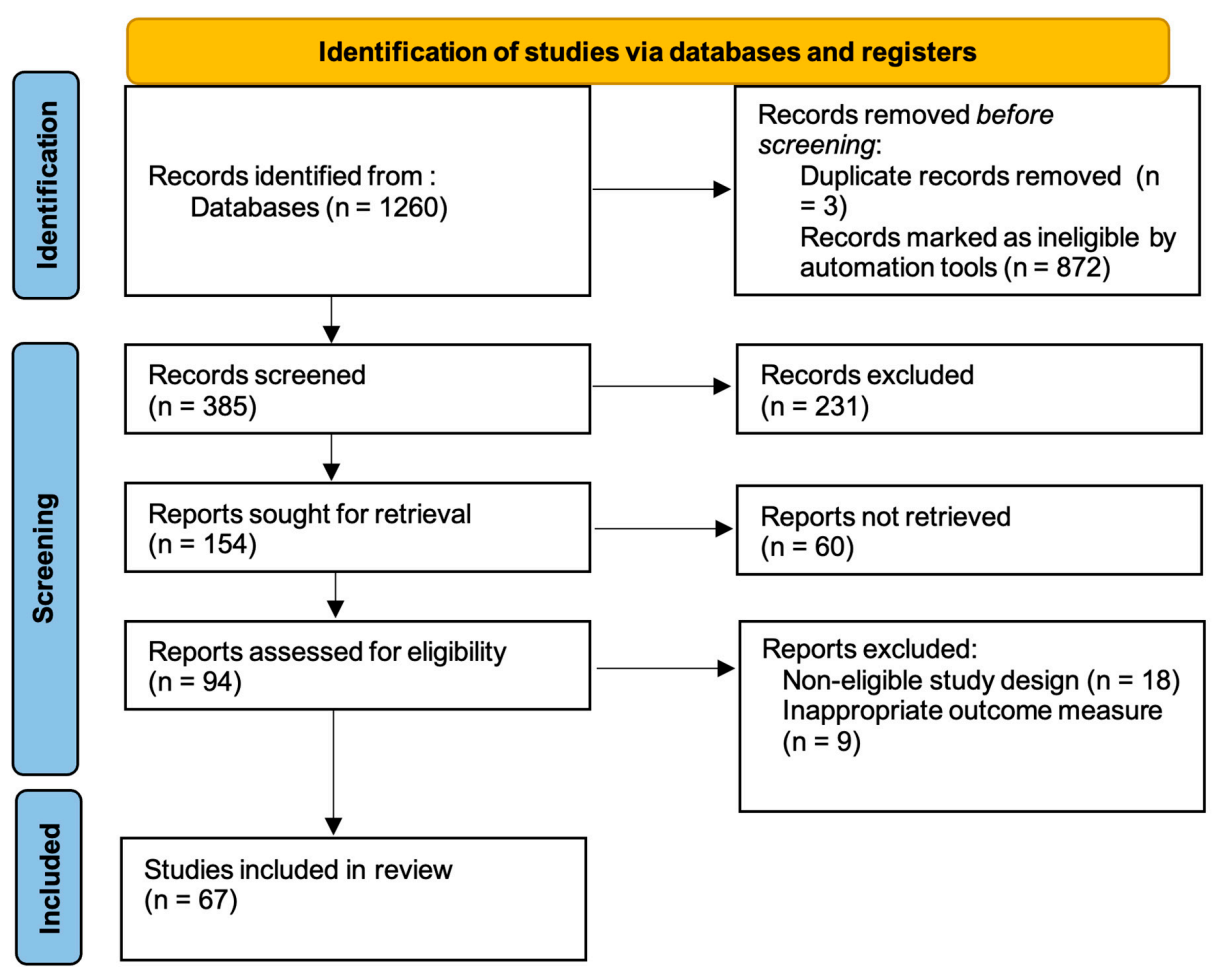
PRISMA Flowchart.

To identify relevant studies on regenerative therapies in periodontology, a manual literature search was conducted in the PubMed Central and Scopus databases. The search was restricted to articles published between December 1, 2015, and July 10, 2025, and included only human studies classified as Clinical Trials or Randomized Controlled Trials (RCTs). The following Boolean search strategy was used: (PERIODONTIUM OR PERIODONTAL OR PERIODONTITIS) AND (GTR OR BONE REGENERATION OR BONE GRAFT OR PLATELET CONCENTRATE OR PRP OR PRF OR I-PRF OR A-PRF OR T-PRF OR PLATELET-RICH FIBRIN OR GROWTH FACTORS OR STEM CELLS OR TISSUE ENGINEERING OR SCAFFOLDS OR LASER THERAPY OR LASER), resulting in a total of 1260 clinical trials and randomized controlled trials manuscripts as seen in Figure 1**.** Additional filters were applied to include only articles published in English, available in Open Access, and focused on clinical applications. Animal studies and articles without direct clinical relevance were excluded. After selecting the full free-text manuscripts published in English in the two databases, a total of *385* articles were eligible for the eprocess. The remaining 385 articles were screened manually by two independent reviewers to assess titles and abstracts for eligibility. Of these, 154 full-text reports were sought for retrieval, and 94 were evaluated in detail for methodological relevance to periodontal regenerative therapies. Discrepancies between reviewers were solved through discussion and consensus with a third evaluator. Following the exclusion of studies with non-eligible designs or inappropriate outcome measures, 67 articles were finally included in this narrative review.

The extracted data were synthesized using both tabular and narrative approaches. Key clinical/radiographic parameters at the longest follow-up period were summarized in tables, while qualitative aspects, including biological mechanisms, defect characteristics, and clinical applicability, were discussed narratively.

### 2.3. Risk of Bias

The studies selected for screening were manually evaluated by the authors using Rayyan (Rayyan Systems Inc., Cambridge, MA, USA), a web-based software designed to facilitate review processes. The platform allowed blinded, independent assessment and conflict resolution among reviewers during the risk of bias evaluation.

A total of 67 articles met the inclusion criteria and were analyzed in this review. The included studies mainly consisted of randomized controlled trials and clinical trials published between 2015 and 2025, focusing on regenerative periodontal therapies in human subjects. The selected literature was categorized according to the principal regenerative strategies investigated, including guided tissue and bone regeneration (GTR/GBR), bone grafting techniques, biological therapies such as platelet concentrates, enamel matrix derivatives (EMD), hyaluronic acid (HA), cell-based and tissue engineering approaches, and laser-assisted treatments.

## 3. Results

The main findings for each regenerative approach are summarized in the following subsections and tables.

### 3.1. Guided Tissue Regeneration (GTR)

Periodontal regeneration aims to achieve new bone growth and periodontal ligament (PDL) fibers attached to a newly formed cementum and alveolar bone [18].

Essential components must be present to successfully promote periodontal regeneration: adequate levels of regulatory signals, progenitor cells, a sufficient blood supply, and the proper use of a suitable biomaterial scaffold. The core principle of GTR revolves around selective cell repopulation. Epithelial cells are known to migrate into the periodontal defect, hindering the formation of normal periodontal tissues. This process can be mitigated by a barrier inhibiting epithelial growth and encouraging periodontal regeneration [18,19].

The barriers utilized in guided tissue regeneration possess varying physicochemical properties, and several generations of membranes have been described over time. The first generation includes non-resorbable membranes, whereas the second generation features entirely resorbable membranes [20]. Based on a review of the literature, the focus is on the development of a new generation of membranes, referred to as the third generation, which is capable of releasing growth factors, adhesion factors, or antibiotics in a controlled manner [21,22].

Collagen membranes are among the most widely used biomaterials, both in guided tissue regeneration (GTR) and guided bone regeneration (GBR), due to their excellent biocompatibility, ease of handling, and favorable resorption characteristics. Derived primarily from bovine or porcine sources, these membranes act as barriers to prevent epithelial downgrowth into the defect site, thus facilitating selective repopulation by periodontal ligament and bone-forming cells [20,23,24].

Modified perforated membranes (MPMs) represent an innovative approach in GTR. They are designed to have micropores and microperforations that allow gingival mesenchymal stem/progenitor cells (GMSCs) and periosteum-derived stem/progenitor cells (PDPCs), growth and differentiation factors to participate in the supracrestal regeneration of intrabony defects [24].

Polytetrafluoroethylene (PTFE) membranes, especially in their expanded form (e-PTFE), represent the first generation of barrier materials successfully used in guided bone regeneration (GBR). As a synthetic polymer, they are known for their biocompatibility, resistance to enzymatic degradation, and minimal immunogenicity. Despite their effective barrier function and structural stability, early studies reported limitations related to membrane collapse under soft tissue pressure. Therefore, PTFE membranes were reinforced with titanium frameworks to improve their dimensional stability and performance [25].

Several clinical studies have compared the effectiveness of different membrane types and graft combinations in regenerative therapies. These investigations have evaluated various outcomes, including clinical attachment level (CAL) gain, probing depth (PPD) reduction, radiographic defect fill, and bone augmentation stability. Table 1 summarizes selected comparative studies that highlight the clinical performance of modified versus conventional membranes, resorbable versus non-resorbable membranes, and different combinations of grafting materials.

### 3.2. Bone Grafting

While guided tissue regeneration primarily aims at restoring the soft periodontal tissues, such as the gingiva and periodontal ligament, Guided Bone Regeneration (GBR) is focused on hard tissue regeneration, with the primary objective of augmenting alveolar bone volume to facilitate optimal bone healing and support for future implant placement or periodontal stability. GBR is widely used for treating intrabony defects and is known for its reliable bone formation results, low resorption rates over time, easy defect filling and contouring, and relatively few surgical complications. The technique involves applying different bone grafts in combination with a barrier membrane, making it a standard approach that contributes to consistent clinical success. A variety of graft materials are available, including autografts, allografts, and xenografts [32]. Since GBR aims to regenerate a single tissue, it is, in theory, easier to achieve than GTR, which seeks to regenerate multiple tissues at once.

*Autografts*, harvested from the patient’s own body, possess osteogenic, osteoinductive, and osteoconductive properties. However, limitations such as donor site morbidity and limited graft volume may restrict their use [33].

*Allografts*, obtained from human donors, offer the advantage of eliminating a second surgical site while maintaining favorable osteoconductive and, in some forms, osteoinductive potential [34]. Freeze-dried bone allograft (FDBA) is a widely used allogenic graft material in periodontal and implant-related regenerative procedures. It is derived from human donor bone and processed through a freeze-drying technique that removes cellular components while preserving the mineral content and overall structure. FDBA is primarily osteoconductive, providing a biocompatible scaffold that facilitates the migration, attachment, and proliferation of osteogenic cells from surrounding bone and soft tissue. It offers greater structural stability but lacks significant osteoinductive potential like demineralized freeze-dried bone allograft (DFDBA). DFDBA is known for its osteoinductive properties because it contains several growth and differentiation factors, particularly bone morphogenetic proteins (BMPs) 2, 4, and 7, which are believed to enhance periodontal healing and regeneration by contributing to PPD reduction, CAL gain, and increased bone fill [35].

*Xenografts*, derived from non-human species (commonly bovine and porcine sources), are widely used due to their availability, biocompatibility, and slow resorption rate, which provides long-term scaffold support. Deproteinized bovine bone mineral (DBBM) is one of the most extensively studied and commonly used xenogenic graft materials in periodontal and implant therapy. Its biomimetic structure, featuring both micro- and macro-porosity resembling human cancellous bone, supports early vascular ingrowth and facilitates the recruitment of osteogenic progenitor cells. In addition, DBBM exhibits mechanical properties that enable space maintenance and serve as a scaffold for new bone formation [32,36]. In recent years, demineralized porcine bone matrix (DPBM) has emerged as an alternative xenograft, offering structural and compositional similarities to human bone, and has shown promising results in periodontal regenerative procedures [37,38].

According to a study [39], GBR with non-resorbable membranes can provide enhanced dimensional stability of the augmented bone compared to resorbable membranes. Nevertheless, the advantages may be counterbalanced by an associated increase in soft tissue thickness observed with the use of resorbable membranes.

Several clinical studies have explored the effectiveness of different combinations of graft materials and membranes in guided bone regeneration, particularly in the context of simultaneous implant placement. Table 2 summarizes the outcomes of recent comparative trials, various types of xenografts, and the adjunctive use of concentrated growth factors.

According to the current European Federation of Periodontology (EFP) clinical practice guideline, the use of barrier membranes, with or without the addition of bone grafting materials, remains the recommended approach for the treatment of intrabony periodontal defects. These techniques provide space maintenance and promote selective cell repopulation, leading to improved clinical attachment gain and bone fill compared with open flap debridement alone [11].

### 3.3. Biological Therapies

Beyond the mechanical principles of guided bone and tissue regeneration, contemporary periodontal therapy has shifted toward biologically based approaches that enhance the body’s intrinsic regenerative capacity.

#### 3.3.1. Platelet Concentrates (PC)

Blood plays a crucial role in the natural wound-healing process by supplying the necessary components for tissue regeneration. Platelet concentrates (PC) are autologous products that are bioactive and composed of platelets, sometimes leukocytes, and are rich in growth factors and fibrin, which serves as the supporting matrix. Over time, several types of autologous platelet derivatives have been described, from the first generation represented by Platelet-Rich Plasma (PRP) to the second generation, Platelet-Rich Fibrin (PRF), and the third generation, which includes advanced Platelet-Rich Fibrin (A-PRF), injectable platelet-rich fibrin (I-PRF), and titanium Platelet-Rich Fibrin (T-PRF). All of these are obtained from the patient’s venous blood, with the differences mainly represented by the working protocols used to obtain them [40,41,42].

Autologous platelet-rich plasma (PRP) is a concentrated mixture of growth factors derived from platelets: platelet-derived growth factor (PDGF) and transforming growth factor-β (TGF-β) within their alpha granules. As a high-concentration platelet suspension, PRP is an outstanding source of growth factors, especially PDGF and TGF-β. PDGF, recognized as a biological modifier, plays a vital role in the healing of bone and the periodontium by influencing essential cellular processes. PRP is typically prepared using a double centrifugation technique. Usually, 20 mL of venous blood is drawn into vacuum tubes containing sodium citrate to prevent premature platelet activation. An initial centrifugation at 1500 rpm for 10 min enables the separation of red blood cells (RBCs) from the plasma, which contains the majority of white blood cells (WBCs) and platelets. The resulting plasma layer, along with a minimal volume of RBCs, is then transferred to a sterile tube and subjected to a second centrifugation at 2500 rpm for 10 min. After this step, the upper fraction of platelet-poor plasma (PPP) is removed, and the lower platelet-rich portion is gently homogenized [43].

Platelet-Rich Fibrin (PRF) has emerged as a significant advancement in regenerative medicine and periodontal therapy since 2001. Unlike Platelet-Rich Plasma (PRP), which often releases growth factors faster and for a limited duration, PRF offers a more sustainable release mechanism. The preparation of PRF is less time-consuming, requiring no use of anticoagulants or additional biochemical handling, thus minimizing procedural complexity. Moreover, PRF is less technique-sensitive, reducing the risk of variability in clinical outcomes associated with operator-dependent factors [44,45].

PRF is an autologous biomaterial that creates a three-dimensional fibrin matrix, providing a reservoir for platelets and various cytokines that are crucial for wound healing and tissue regeneration [46]. Autologous platelet derivatives, including platelet-rich fibrin (PRF), have progressively been utilized to support soft tissue repair and accelerate bone regeneration by promoting key biological processes such as chemotaxis, angiogenesis, cellular proliferation, and stem cell activation [28].

Since most platelet concentrates are solid or in gel form, they are unsuitable for injection. This limitation has been addressed by the emergence of a new platelet concentrate type, I-PRF, produced using an alternative centrifugation protocol. It is well established that periodontal treatment yields less favorable outcomes in smokers compared to non-smokers. However, evidence from the literature indicates that the adjunctive use of injectable platelet-rich fibrin (I-PRF) in combination with scaling and root planing (SRP), particularly when the platelet concentrate was reapplied on the seventh day, results in greater probing depth reduction and improved clinical attachment gain [47].

Titanium-prepared platelet-rich fibrin (T-PRF) is a third-generation of platelet concentrate, and its characteristics are the formation of a thicker fibrin layer, a longer absorption time, and increased capacity of osseointegration, thus promoting periodontal regeneration [48].

Recent studies have assessed the effectiveness of platelet concentrates as carriers for specific adjuvant substances, such as antibiotics [49]. The application of PRF, with or without Metronidazole, within periodontal pockets was associated with favorable clinical outcomes in the treatment of moderate periodontitis [50].

Numerous randomized clinical trials have evaluated the adjunctive use of platelet concentrates in different periodontal and peri-implant therapies, demonstrating enhanced wound healing and clinical attachment gain compared with conventional treatments, as summarized in Table 3.

Although the current EFP S3 clinical practice guideline does not provide explicit recommendations regarding the use of platelet-derived products in periodontal regeneration, it emphasizes the importance of meticulous flap design to optimize healing outcomes. In particular, the guideline recommends the use of minimally invasive surgical approaches that preserve the interdental papilla as much as possible, thus maintaining vascular integrity and reducing postoperative recession. Furthermore, flap elevation should be limited to what is strictly necessary to allow proper access to the defect, in order to promote early wound stability [11].

#### 3.3.2. Enamel Matrix Derivatives (EMD)

Beyond platelet-derived biomaterials, enamel matrix derivatives represent another category of biologically active molecules with well-documented effects on periodontal wound healing and regeneration.

Enamel Matrix Derivatives (EMD) have been used for over 20 years and are now regarded as some of the standard materials in periodontal regeneration. They have been utilized as an adjunctive agent during surgical procedures to enhance both soft and hard tissue regeneration, as demonstrated by PPD reduction and CAL gain [42,64]. EMD plays a key role in cementum formation and periodontal ligament regeneration and can be an alternative to barrier membranes in certain cases. However, its use requires careful handling, as blood contamination can negatively impact the adhesion and growth of periodontal ligament cells [65].

In addition, the EFP S3 clinical practice guideline recommends the use of enamel matrix derivatives for the management of residual periodontal pockets associated with intrabony defects [11].

According to the AAP, both platelet concentrates and enamel matrix derivatives (EMD) are classified as periodontal biologics, with well-documented effectiveness in the treatment of intrabony defects. Clinical evidence consistently shows that these biologic agents provide significant clinical improvements, and their overall benefit–to–risk profile remains highly favorable, particularly when applied under appropriate surgical conditions and within minimally invasive regenerative protocols [66].

#### 3.3.3. Hyaluronic Acid (HA)

In addition to protein-based agents such as EMD, polysaccharide-derived materials have also been explored for their regenerative potential. Among these, hyaluronic acid has gained attention as a naturally occurring component of the extracellular matrix that modulates inflammation, angiogenesis, and cell proliferation during periodontal healing.

Hyaluronic acid (HA) has emerged as a promising biomaterial used for periodontal regeneration, due to its distinct physicochemical and biological characteristics. Its hygroscopic and viscoelastic properties facilitate water retention, metabolite transport, and structural support of tissues through interactions with cellular and extracellular matrix components. HA exhibits a range of therapeutic effects, including antimicrobial activity and osteoinductive potential, both of which contribute to enhanced periodontal regeneration. Furthermore, it inhibits the excessive proliferation of the gingival epithelium and supports the adhesion and proliferation of periodontal ligament cells by activating CD44 receptors. The biological activity of HA is modulated by its molecular weight and concentration, which vary depending on the target cell type and receptor expression. Therefore, hyaluronic acid is considered a key adjunct in regenerative periodontal therapy owing to its ability to modulate inflammation, granulation tissue formation, wound remodeling, and clot stabilization [65]. A recent clinical study comparing hyaluronic acid with enamel matrix derivative for the treatment of intrabony periodontal defects reported that both treatment modalities led to clinical improvements over 24 months. Although EMD achieved a significantly greater reduction in probing depth compared to HA, the clinical relevance of this difference remains uncertain. These findings suggest that HA may serve as a promising alternative in regenerative periodontal surgery. However, further randomized clinical trials with larger sample sizes, as well as histological investigations, are required to understand the nature of periodontal healing associated with hyaluronan application [67].

Table 4 illustrates key clinical studies that have assessed the outcomes regarding HA and EMD in periodontal regenerative therapies. The comparisons include various combinations of biomaterials and surgical techniques, with a focus on outcomes such as CAL gain, ECM remodeling, and postoperative discomfort. While some studies demonstrated statistically significant advantages for EMD [37,64,67], others reported similar clinical results with HA [65,68].

### 3.4. Tissue Engineering

Tissue engineering has become a promising approach for periodontal regeneration, especially through adult mesenchymal cells (MSCs). These undifferentiated cells, present in specific adult tissues and organs, can self-renew and differentiate into a limited range of cell types. Due to their embryological origin and postnatal development, dental tissues are a rich source of MSCs. Successfully isolated from structures such as exfoliated deciduous teeth, apical papilla, dental follicle, periodontal ligament, and dental pulp, stem cells are particularly notable in dental pulp. Human dental pulp stem cells (DPSCs) are especially significant because of their accessibility, shared embryonic origin and antigenic profile with periodontal stem cells, and their ability to develop into periodontal-specific cell lineages. Additionally, DPSCs show advantageous features like long-term viability, biomaterial compatibility, and stability during cryopreservation [72].

Progenitor cells derived from periodontal ligament (PDL-derived MSCs) exhibit the potential to differentiate into multiple periodontal-related lineages, such as cementoblasts, fibroblasts, and osteoblasts, making them ideal candidates for comprehensive periodontal regeneration [19]. A controlled clinical pilot study evaluated the safety and potential of PDL-derived MSCs delivered through hydroxyapatite-collagen scaffolds for treating intrabony defects. Results confirmed the method’s safety, showing no rise in adverse effects compared to standard regenerative procedures. Although the study did not reveal statistically significant improvements in CAL gain or PPD reduction, positive trends were noted in the group receiving cell-based therapy [73].

Additionally, mesenchymal stem cells obtained from peripheral blood (PB-MSCs) have gained attention due to their minimally invasive collection process and autologous nature. Unlike bone marrow-derived MSCs (BM-MSCs), which require more invasive harvesting techniques and carry risks such as pain, bleeding, and neurovascular complications, PB-MSCs offer a safer and more patient-friendly alternative for future clinical applications [74].

### 3.5. Laser Therapy

Laser technology has been widely used in dentistry for over four decades, marking a significant evolution from diagnostic to therapeutic applications. Within periodontology, lasers represent a minimally invasive and precise treatment modality capable of promoting decontamination, coagulation, and biostimulation while minimizing tissue trauma. Their versatility allows for use in both non-surgical and surgical periodontal therapy, aiming to enhance healing, reduce inflammation, and improve patient comfort [75].

#### 3.5.1. Antimicrobial Photodynamic Therapy (aPDT)

Antimicrobial photodynamic therapy (aPDT) has emerged as a promising adjunctive approach to conventional mechanical periodontal debridement. It involves the use of a photosensitizing agent, selectively taken up by bacterial cells, followed by targeted light irradiation, which activates the photosensitizer to produce cytotoxic singlet oxygen species. These reactive oxygen species exert antimicrobial effects by damaging cellular components, with a low risk of inducing bacterial resistance. Beyond the oxidative mechanism, indocyanine green (ICG), a photosensitizer widely used as a perfusion imaging agent, has demonstrated photothermal effects when activated by near-infrared (NIR) laser irradiation [76]. Extracellular metalloproteinases (MMPs), especially 8 and 9 (MMP-8 and MMP-9), play a major role in periodontal destruction, being observed at significantly higher levels in the gingival crevicular fluid (GCF) of patients with periodontal disease compared to healthy individuals. According to the literature, the systemic use of antibiotics, such as amoxicillin and metronidazole, seems to be more effective in reducing the GCF MMP-8 levels compared to the adjunctive use of PDT [77].

#### 3.5.2. Low-Level Laser Therapy (LLLT)

Another adjunctive approach that may enhance the effectiveness of periodontal therapy is Low-level Laser Therapy (LLLT), also known as photobiomodulation therapy. This technique utilizes infrared or near-infrared light to produce analgesic, anti-inflammatory, and biostimulatory effects at the tissue level. LLLT has been reported to reduce inflammation, promote wound healing, modulate immune responses, relieve pain, and stimulate the growth of new cells and tissues [78].

At the subcellular level, LLLT primarily targets the mitochondria, where cytochrome c oxidase (CCO) functions as the main photoreceptor. Activation of CCO by laser light leads to enhanced mitochondrial membrane potential, increased production of adenosine triphosphate (ATP), and transfer of cytochrome C to molecular oxygen. These processes contribute to the upregulation of cellular metabolism and regeneration [79].

Although microbiological analyses have shown a slightly greater reduction in key periodontal pathogens such as Porphyromonas gingivalis and Treponema denticola following LLLT, these differences are not statistically significant. Furthermore, there is currently no standardized protocol regarding optimal parameters or treatment duration for LLLT in periodontal therapy, which limits its widespread adoption and consistent application in clinical settings [80].

#### 3.5.3. Laser-Assisted New Attachment Procedure (LANAP)

LANAP is an emerging laser-based surgical method designed to promote periodontal tissue regeneration through a minimally invasive approach. It uses a pulsed Nd: YAG laser (wavelength 1064 nm) following a carefully sequenced, multi-step protocol. First, laser irradiation selectively removes the diseased pocket epithelium and lowers the microbial population, targeting periodontopathogenic bacteria and heat-sensitive endotoxins while sparing nearby healthy tissues. This is followed by thorough mechanical debridement and root planing to eliminate residual calculus and contaminated cementum. A second laser application then encourages stable fibrin clot formation within the sulcus, creating a sealed environment that prevents epithelial downgrowth and promotes the formation of new connective tissue attachment. Histologic studies have shown evidence of new cementum, bone, and periodontal ligament formation after LANAP therapy, indicating a true regenerative potential. Clinically, the procedure has been linked to reductions in probing depth and improvements in clinical attachment levels; however, the current evidence base is limited due to the scarcity of randomized controlled clinical trials evaluating its biochemical and radiographic outcomes [78].

According to the EFP S3 clinical practice guideline, the routine use of lasers and antimicrobial photodynamic therapy (aPDT) as adjuncts to scaling and root planing is not suggested. This position is primarily based on the unclear or high risk of bias present in many of the available clinical trials, as well as the considerable heterogeneity in laser parameters, wavelengths, and treatment protocols. Moreover, the current evidence fails to demonstrate consistent or clinically meaningful additional benefits when aPDT is combined with conventional non-surgical periodontal therapy [11]. According to the AAP consensus, the combination of scaling and root planing (SRP) with antimicrobial photodynamic therapy (aPDT) is recommended with a moderate level of certainty for both aggressive and chronic periodontitis. Meta-analyses indicate that SRP + aPDT provides modest additional clinical improvements compared with SRP alone, particularly in deep periodontal pockets (≥7 mm). However, the available evidence does not clearly establish the clinical relevance of these incremental benefits, and none of the included studies reported treatment cost analyses [81].

Several studies have compared the clinical outcomes of laser-assisted therapies with conventional non-surgical or surgical periodontal treatments, reporting variable results in terms of probing depth reduction, clinical attachment gain, and microbial load, as shown in Table 5.

## 4. Discussion

This review synthesized clinical evidence from 67 randomized and controlled clinical trials evaluating a wide range of regenerative periodontal approaches.

### 4.1. Quality of Evidence and Variability Among Studies

The available evidence remains heterogeneous in terms of study design, defect morphology, biomaterial characteristics, and follow-up duration. Although most included trials reported clinically favorable improvements in PPD reduction and CAL gain, many presented unclear randomization procedures, limited masking of examiners, or small sample sizes—factors that increase the risk of bias. Moreover, follow-up periods ranged widely, and long-term stability of regenerative outcomes remains insufficiently documented. As wound shrinkage typically continues for several months postoperatively, short-term evaluations may not accurately reflect the final outcome of regenerative procedures. Pain assessment conducted one day after surgery in several studies may also introduce reporting bias due to the early inflammatory phase. Consequently, results should be interpreted with caution, and future multicenter trials with standardized protocols are needed to strengthen the evidence base.

### 4.2. Patient-Related and Microbiological Factors

Systemic conditions, behavioral factors, and microbiological profiles continue to play an essential role in treatment outcomes. Within the studies included in this review, some of the regenerative protocols combined platelet concentrates with adjunctive antibiotic therapy: clindamycin and metronidazole, to enhance antibacterial effects and improve early wound healing. According to the EFP S3 clinical practice guideline, adjunctive antibiotics may be considered in carefully selected cases with high microbial load; however, harm versus benefit considerations must be carefully evaluated, as overuse may contribute to antimicrobial resistance and unnecessary patient exposure. For this reason, antibiotics should be restricted to well-justified clinical scenarios and integrated within an evidence-based periodontal treatment plan [11].

### 4.3. Surgical Techniques and Operator-Dependent Factors

The EFP guideline emphasizes that regenerative periodontal procedures should be performed only by clinicians with advanced training, given the technical complexity of these interventions. Minimally invasive surgical techniques (MIST, M-MIST) are strongly favored, as they preserve the interdental papilla, maintain vascular supply, and enhance soft tissue stability during healing. Excessive flap elevation should be avoided to reduce trauma and minimize postoperative shrinkage. Consistent with the reviewed literature, surgical precision, including flap management, defect debridement, graft stability, and wound closure, often exerts a greater influence on outcomes than the choice of biomaterial itself. Therefore, operator skill remains a critical determinant of regenerative success [11,12].

### 4.4. Clinical Implications, Limitations, and Generalizability

The findings of this review suggest that biologically active materials such as EMD, PRF, and hyaluronic acid can achieve clinically favorable outcomes in selected intrabony defects. In the limited number of studies comparing hyaluronic acid with enamel matrix derivatives, both materials demonstrated significant improvements in CAL and PPD, although no statistically significant intergroup differences were observed. Notably, EMD is highly sensitive to blood contamination, whereas hyaluronic acid maintains its stability and adhesive properties even in moist conditions—an advantage in situations where complete hemostasis cannot be consistently achieved. Future trials should compare different HA formulations to reduce heterogeneity and strengthen clinical recommendations [65,68].

Despite these encouraging findings, the generalizability of results is limited. Many included studies were conducted in academic centers with highly controlled environments, which may not fully reflect routine clinical practice. Several factors—such as defect morphology, periodontal phenotype, smoking status, or plaque control—were inconsistently reported across studies. Additionally, this review was restricted to English-language, open-access databases, which may have introduced publication bias and excluded potentially relevant evidence. These methodological limitations underscore the need for more standardized, rigorously designed clinical trials.

### 4.5. Alignment with Guidelines and Future Directions

Examining current clinical guidelines reveals that many regenerative techniques discussed, such as laser therapies, antimicrobial photodynamic therapy, tissue-engineered constructs, and advanced biomaterials, remain experimental and are not suggested for routine use [11]. This cautious approach is justified due to the unclear or high bias risk in many studies and the lack of consistent, clinically significant benefits over established options like GTR/GBR, EMD, or platelet concentrates. Future research should focus on long-term, multicenter randomized controlled trials with standardized protocols, defect-specific algorithms, and clear outcome measures. Comparing biologics like EMD, hyaluronic acid, and platelet concentrates is crucial for forming clinical guidelines. Additionally, specific studies point to key areas for further investigation, such as testing CXP membranes in large augmentation procedures, evaluating clindamycin-loaded I-PRF with extended follow-ups and larger samples, and assessing long-term effects of subgingival i-PRF in smokers via multicenter trials [20,62]. Standardizing next-generation platelet concentrates and differentiating outcomes based on defect types can improve reliability. Employing split-mouth designs could reduce variability and enable direct comparisons between T-PRF, L-PRF, and natural healing [54]. Ultimately, standardized high-quality protocols will improve treatment predictability, facilitate evidence-based decisions, and ensure clinical practices align with current AAP/EFP guidelines.

## 5. Conclusions

Periodontal regeneration remains a central objective in modern periodontal therapy, aiming to restore the form and function of lost supporting tissues. Over the past decade, advances in biomaterials and surgical protocols have contributed to more favorable clinical outcomes. GTR, PC, EMD, and HA have each demonstrated clinically favorable outcomes in selected cases, in accordance with current evidence-based guidelines. However, the overall predictability of these regenerative modalities is still limited by heterogeneity in study design, defect characteristics, and follow-up duration. Further long-term randomized clinical trials are required to validate these findings and to develop standardized, predictable treatment protocols aligned with the evidence-based recommendations.

## Figures and Tables

**Table 1:** Comparative clinical outcomes using different membrane types and graft combinations. Abbreviations: 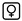
—women, 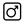;—men, PTFE—polytetrafluoroethylene, d-PTFE—dense polytetrafluoroethylene, e-PTFE—expanded polytetrafluoroethylene, VBG—vertical bone gain, MPM—modified perforated membrane, CM—collagen membrane, CAL—clinical attachment loss, PPD—periodontal pocket depth, RDD—radiographic defect depth, CXP—creos xenoprotect, PRF—platelet-rich fibrin L-PRF—leukocyte platelet-rich fibrin, GR—gingival recession, ABG—autologous bone graft, CAF—coronally advanced flap, CGF—concentrated growth factor, CTG—connective tissue graft, KTT—keratinized tissue thickness, KTW—keratinized tissue width, RC—recession coverage, GTR—guided tissue regeneration.

Study	No. of Patients	Group A	Group B	Outcome	Conclusions
Alessandro, C. et al. [23]	40 (27 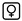 and 13 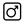 )	d-PTFE titanium-reinforced membrane (*n* = 20)	titanium mesh (Ti mesh) + cross-linked CM (*n* = 20)	VBG at 9 months: 4.2 ± 1 mm (Group A) vs. 4.1 ± 1 mm (Group B)	Similar VBG were achieved in both groups.
Bartłomiej, G. et al. [24]	15 (10 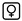 and 5 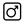 )	xenogenic graft + MPM (*n* = 15)	xenogenic graft + CM (*n* = 15)	PPD reduction at 12 months: 4 ± 2.1 mm vs. 3.5 ± 1.2 mm CAL gain 4.7 ± 2.1 vs. 4.3 ± 1.3	Both GTR with MPM and CM membranes led to significant clinical improvements at 12 months.
Bastian, W. et al. [20]	49 (20 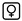 and 29 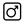 )	xenogenic graft + CXP (*n* = 24)	xenogenic graft + CM (*n* = 25)	VBG at 6 months: 4.1 ± 2.2 mm (Group A) vs. 3.3 ± 2.8 mm (Group B)	Both collagen membranes resulted in safe bone augmentation.
Giuseppe, B. et al. [26]	64	L-PRF+ ABG (*n* = 32)	CM + ABG (*n* = 32)	PPD reduction at 12 months: 4.28 ± 1.22 mm (Group A) vs. 4.5 ± 1.37 mm (Group B) CAL gain 3.44 ± 0.8 mm (Group A) vs. 3.75 ± 1.37 mm (Group B)	Both techniques were found to be effective in the treatment of intrabony defects.
Istvan A, U. et al. [27]	30 (21 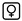 and 9 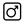 )	perforated titanium-reinforced d-PTFE mesh + CM (*n* = 15)	PTFE alone (*n* = 15)	VBG at 9 months: 4.11 ± 2.69 mm (Group A) vs. 4.47 ± 2.05 mm (Group B)	Both techniques led to comparable outcomes for VBG
Ramya Naga Shivani, C. et al. [28]	35 (17 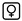 and 18 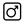 )	non-submerged implants with a PRF membrane (*n* = 18)	non-submerged implants alone (*n* = 17)	KTT at 6 months: 0.97 ± 0.12 mm (Group A) vs. KTT 0.59 ± 0.57 (Group B) KTW at 6 months: 1.25 ± 0.67 mm (Group A) vs. 0.59 ± 0.36 mm (Group B)	The PRF membrane enhances peri-implant tissue wound healing, with gains in soft tissue width and thickness around non-submerged implants
Serap Karakış, A. et al. [29]	19 (8 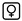 and 11 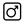 ), 74 Miller Class I GRs	CAF + CGF membrane (*n* = 37)	CAF + CTG (*n* = 37)	KTT at 6 months: 1.63 ± 0.31 mm (Group A) vs. 1.38 ± 0.34 mm (Group B) KTW at 6 months: 3.03 ± 1.02 mm (Group A) vs. 3.57 ± 1.14 (Group B)	CTG is superior to CGF with CAF for increasing KTT, KTW, and RC. CGF may be preferable due to decreased postoperative pain
Tobias, B. et al. [30]	23 (12 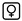 and 11 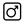 )	xenogenic graft + resorbable CM	xenogenic graft + non-resorbable ePTFE-membrane	Interproximal marginal bone levels at 3 years: 0.19 ± 0.21 mm (Group A) vs. 0.16 ± 0.10 mm (Group B)	Stable interproximal bone levels and healthy tissues can be obtained with membranes up to 3 years
Santhi P, P. et al. [31]	15	CAF (*n* = 15)	CAF + CM (*n* = 15)	Root coverage percentage at 6 months: 71.6 ± 5.9% (Group A) vs. 73.13 ± 25.5% (Group B)	Integrating this approach with placing a bio-absorbable membrane does not seem to improve the results following surgical treatment of Miller’s Class I and II recessions

**Table 2:** Comparative outcomes of GBR techniques using different graft materials. Abbreviations: 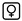
—women, 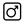;—men, GPCGF—gel phase concentrated growth factor, DBBM—deproteinized bovine bone mineral, LPCGF—liquid phase concentrated growth factor, GBR—guided bone regeneration, CM—collagen membrane, e-PTFE—expanded polytetrafluoroethylene, DPBM—deproteinized porcine bone material, HT- horizontal thickness, SBS—synthetic bone substitute.

Study	No. of Patients	Group A	Group B	Group C	Outcome	Conclusions
Lingshan, Z. et al. [32]	57 (28 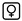 and 29 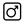 )	GPCGF + DBBM (*n* = 19)	LPCGF + DBBM (*n* = 19)	DBBM alone (*n* = 19)	Buccal lateral bone thickness at 6 months after surgery: 2.74 ± 0.91 mm (Group A) vs. 2.98 ± 1.23 mm (Group B) vs. 2.87 ± 0.87 mm (Group C)	GPCGF + DBBM enhances the outcomes of simultaneous GBR in implant therapy by reducing bone resorption, promoting bone regeneration, and mitigating postoperative complications compared to DBBM alone
Strauss, F.J. et al. [39]	27 (14 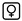 and 13 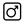 )	xenogenic graft + CM (*n* = 13)	xenogenic graft + titanium reinforced e-PTFE membrane (*n* = 14)	-	Bone thickness difference at 6 months: 0.7 ± 0.7 mm (Group A) vs. 0.1 ± 0.3 mm (Group B)	e-PTFE membrane seems to provide greater dimensional stability compared to CM
Ji-Young, J. et al. [38]	69	particulate DPBM (*n*= 34)	cross-linked collagenated DPBM (*n* = 35)	-	HT at 4 months: 1.96 ± 0.79 mm (Group A) vs. 2.43 ± 1.02 mm (Group B)	No significant differences between cross-linked collagenated and particulated DPBMs
Jae-Kook, C. et al. [36]	49 (31 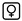 and 18 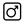 )	SBS + CM (*n* = 24)	DBBM + CM (*n* = 25)	-	Bone height gain at 6 months 3.8 ± 3.7 mm (Group A) vs. 2.6 ± 2.7 (Group B)	The SBS is noninferior to DBBM for simultaneous GBR to implant placement

**Table 3:** Outcomes related to PC use in different periodontal treatments. Abbreviations: 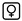
—women, 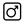;—men, AA—ascorbic acid, ADM—acellular dermal matrix, CAF—coronally advanced flap, OFD—open-flap debridement, PMPR—professional mechanical plaque removal, PRF—Platelet-Rich Fibrin, 2PRF—two layers of stacked PRF membranes, 4PRF—four layers of stacked PRF membranes, A-PRF—advanced platelet-rich fibrin, I-PRF—injectable platelet-rich fibrin, L-PRF—leukocyte platelet-rich fibrin, T-PRF—titanium prepared platelet-rich fibrin, CTG—connective tissue graft, MAGR—multiple adjacent gingival recessions, EMD—enamel matrix derivatives, DFDBA—demineralized freeze-dried bone allograft, MARF—modified apically repositioned flap, CM—collagen membrane, FGG—free gingival graft, CLN—clindamycin, SRP—scaling and root planing.

Study	No. of Patients	Group A	Group B	Group C	Outcome	Conclusions
Anupama, T. et al. [51]	30 (12 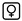 and 18 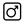 )	CAF + L-PRF (*n* = 15)	CAF + A-PRF (*n* = 15)	-	At 6 months root coverage of 67.2 ± 32.81% (Group A) vs. 81.66 ± 28.21% (Group B)	Both groups may be suggested as treatment for maxillary gingival recessions.
Atacan, Y. et al. [52]	12 (7 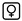 and 5 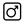 ), 59 defects	CAF + L-PRF (*n* = 30)	CAF + CTG (*n* = 29)	-	PPD at 12 months of 1.60 ± 0.62 mm (Group A) vs. 1.83 ± 0.59 mm (Group B)	L-PRF were equally effective as CTG in treating MAGRs
Csifó-Nagy, B.K. et al. [42]	18 (9 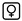 and 9 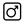 ), 30 sites	A-PRF (*n* = 15)	EMD (*n* = 15)	-	CAL gain at 6 months: 2.33 ± 1.58 mm (Group A) vs. 2.6 ± 1.18 mm (Group B)	A-PRF seems to be as clinically effective as EMD during surgical treatment of intrabony defects
Ozkal Eminoglu, D. et al. [53]	20 (9 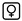 and 11 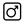 )	OFD + T-PRF (*n* = 20)	OFD alone (*n* = 20)	-	Bone filling ratio at 9 months: 0.27 ± 0.11 (Group A) vs. 0.11 ± 0.04 (Group B)	OFD+ T-PRF group obtained better clinical outcomes
Emad Aldden, A. et al. [54]	30 (14 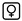 and 16 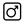 )	T-PRF (*n* = 10)	L-PRF (*n* = 10)	spontaneous healing (*n* = 10)	Radiographic bone density at 4 months: 984.90 ± 14.39 (Group A) vs. 756.80 ± 16.81 (Group B) vs. 730.30 ± 14.14 (Group C)	T-PRF group demonstrated greater preservation of ridge dimensions, higher bone density, improved soft-tissue healing.
Farid, S. et al. [44]	20 (13 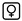 and 7 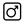 )	PRF membrane (*n* = 10)	ADM (*n* = 10)	-	Gingival thickness after 3 months: 3.300 ± 0.758 mm (Group A) vs. 3.300 ± 0.758 mm (Group B)	Both groups increased gingival thickness
Justina P, L. et al. [45]	30 (14 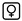 and 16 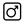 )	PRF matrix (*n* = 15)	CTG (*n* = 15)	-	At 12 months root coverage of 86.20 ± 24.24% (Group A) vs. 91.56 ± 22.32 (Group B)	The study outcomes suggest comparable gains in root coverage and attached gingiva between groups.
Manisha, B. et al. [55]	24	CAF + PRF (*n* = 12)	laser-assisted CAF + PRF (*n* = 12)	-	CAL at 6 months: 3.19 ± 0.89 mm (Group A) vs. 3.26 ± 1.75 mm (Group B)	Both treatments improved clinical parameters post-surgery with no significant differences between groups.
Mashaal Mohammed, A. et al. [35]	20 (13 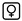 and 7 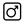 )	I-PRF + DFDBA (*n* = 10)	DFDBA (*n* = 10)	-	CAL gain at 9 months: 3.20 ± 0.63 mm (Group A) vs. 3.70 ± 1.16 mm (Group B)	Addition of I-PRF to DFDBA does not appear to significantly enhance the DFDBA’s regenerative outcomes
Mohamed Abdulhakim, S. et al. [56]	45 (29 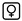 and 16 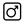 )	PMPR + I-PRF/AA (*n* = 15)	PMPR + I-PRF (*n* = 15)	PMPR alone (*n* = 15)	CAL gain at 6 months: 1.33 ± 0.49 mm (Group A) vs. 1.20 ± 0.56 mm (Group B) vs. 0.93 ± 0.59 mm (Group C)	No intergroup differences were notable regarding clinical improvements
Mohamed Talaat, E. et al. [57]	20 (17 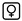 and 3 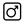 )	OFD + AA/PRF (*n* = 10)	OFD + PRF (*n* = 10)	-	Radiographic linear defect depth reduction at 6 months: 2.29 ± 0.61 mm (Group A) vs. 1.63 ± 0.46 mm (Group B)	Augmenting PRF with AA additionally enhanced gingival tissue gain and radiographic defect fill
Naidu, N.S.S. et al. [58]	12 (4 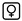 and 8 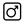 )	DFDBA + PRF (*n* = 12)	DFDBA (*n* = 12)	-	Radiographic bone fill at 9 months: 3.22 ± 1.63 mm (Group A) vs. 3.01 ± 2.37 mm (Group B)	The adjunctive use of PRF did not show any additional benefit in terms of reconstructive output
Parviz, T. et al. [59]	10 (4 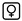 and 6 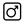 )	MARF + PRF membrane (*n* = 10)	MARF (*n* = 10)	-	Gingival thickness difference at 8 weeks: 0.29 ± 0.15 mm (Group A) vs. 0.11 ± 0.17 (Group B)	Using PRF with the MARF method significantly increased the width and thickness of the gingiva and reduced shrinkage compared to MARF only.
Ramy, M. et al. [60]	30 (18 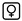 and 12 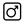 )	OFD (*n* = 10)	PRF alone (*n* = 10)	PRF + CM (*n* = 10)	Radiographic defect height change: 7.25% (Group A) vs. 12.6% (Group B) vs. 32.6% (Group C)	GTR membranes in association with L-PRF improved intrabony defect fill
Rana, C. et al. [61]	22 (12 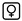 and 10 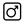 ), 63 teeth	2PRF + CAF (*n* = 21)	4PRF + CAF (*n* = 21)	CTG + CAF (*n* = 21)	CAL at 6 months: 2.86 ± 0.74 mm (Group A) vs. 1.93 ± 0.69 mm (Group B) vs. 1.57 ± 0.71 mm (Group C)	PRF membranes should use as many layers as possible
Sarah, Y. et al. [62]	28 (8 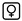 and 20 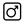 )	CLN/I-PRF + M-MIST (*n* = 14)	I-PRF + M-MIST (*n* = 14)	-	CAL Median (IQR) at 9 months: 1 (0–1) mm (Group A) vs. 1.5 (1–2.25) mm (Group B)	CLN does not appear to further positively impact these observed I-PRF effects
Çağıran Gürbüz, T. et al. [47]	25 (8 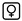 and 17 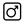 ), 100 sites	SRP + I-PRF (*n* = 50)	SRP + saline (*n* = 50)	-	CAL gain at 3 months: 3.32 ± 0.78 mm (Group A) vs. 2.54 ± 0.80 mm (Group B)	PRF may play a beneficial role in improving the clinical outcomes of nonsurgical periodontal treatment in smokers
Wajeha, A. et al. [63]	16 (10 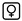 and 6 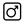 )	I-PRF + FGG (*n* = 16)	FGG alone (*n* = 16)	-	Percentage of root coverage at 6 months: 70.83% ± 20.64% (Group A) vs. 65.62% ± 18.48% (Group B)	The addition of I-PRF improved clinical parameters
Omar, Y.K. et al. [50]	24 (10 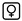 and 14 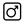 )	SRP + L-PRF/Metronidazole (*n* = 12)	SRP + L-PRF (*n* = 12)	-	CAL percentage change at 6 months: 87.96% (Group A) vs. 75.19% (Group B)	The combination of MTZ with L-PRF was superior to L-PRF alone in managing periodontal diseases

**Table 4:** Comparative studies evaluating the use of enamel matrix derivative and hyaluronic acid in periodontal regeneration. Abbreviations: 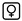
—women, 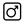;—men, HA—hyaluronic acid, EMD—enamel matrix derivative, NT—no treatment, ECM—extracellular matrix, G- gingival, SRP—scaling and root planing, CAL- clinical attachment level, DPBM—deproteinized porcine bone material, xHyA—cross-linked hyaluronic acid, MIST—minimally invasive surgical technique, M-MIST—modified minimally invasive surgical technique, MPP—modified papilla preservation technique, SPP—simplified papilla preservation technique.

Study	No. of Patients	Group A	Group B	Outcome	Conclusion
Andrea, P. et al. [67]	32 (17 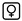 and 15 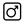 )	HA (*n* = 16)	EMD (*n* = 16)	CAL at 24 months: 5.19 ± 1.42 mm (Group A) vs. 4.44 ± 1.03 mm (Group B)	EMD resulted in statistically significantly higher reduction values compared with HA, the clinical relevance of this difference remains unclear.
Andrea, P.; Lorenzo, M. et al. [69]	8 (4 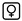 and 4 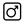 )	HA (*n* = 8)	NT (*n* = 8)	Early wound healing score (EHS) at 1 week: 10(IQR:0) (Group A) vs. 9(IQR:2) (Group B)	HA application resulted in an enhancement of ECM remodeling and collagen maturation, that could act as key drivers of the early wound healing of G tissues
Christian, W. et al. [64]	22 (8 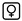 and 3 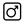 ), 89 sites	SRP + EMD (*n* = 45)	SRP alone (*n* = 44)	CAL gain at 6 months: 1.13 ± 1.58 mm (Group A) vs. 0.47 ± 1.06 (Group B)	EMD in addition to SRP showed an improvement of CAL gain.
Jae-Hong, L. et al. [37]	42 (22 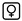 and 20 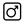 )	DPBM + EMD (*n* = 20)	DPBM alone (*n* = 22)	CAL at 24 months: 6.9 ± 0.86 mm (Group A) vs. 7 ± 0.9 mm (Group B)	The adjunctive use of EMD significantly reduced the postoperative discomfort compared to DPBM alone group.
Jae-Hong, L.; Seong-Nyum, J. [70]	34 (11 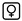 and 12 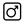 )	DPBM + EMD (*n* = 16)	DPBM alone (*n* = 18)	CAL at 4 years: 6.6 ± 1.0 mm (Group A) vs. 6.6 ± 0.8 (Group B)	No additional clinical and radiographic benefits were observed with the adjunctive use of EMD
Rodríguez-A, M. et al. [65]	53 (28 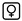 and 25 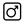 )	HA (*n* = 27)	EMD (*n* = 26)	CAL gain at 18 months: 3.43 ± 1.62 mm (Group A) vs. 3.50 ± 1.81 (Group B)	HA, when used at the right concentration and in suitable cases, shows outcomes comparable to EMD, making it a viable option for regular clinical use.
Vela, O.-C. et al. [68]	54 (31 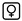 and 22 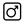 )	xHyA (*n* = 27)	EMD (*n* = 27)	CAL gain at 6 months: 3.18 ± 1.49 mm (Group A) vs. 2.58 ± 1.39 mm (Group B)	xHyA may serve as a promising alternative to EMD for treating intrabony periodontal defects, particularly due to its ease of surgical handling and comparable regenerative potential.
Peter, W. et al. [71]	47 (28 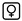 and 19 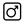 )	MIST/M-MIST + EMD (*n* = 23)	MPP/SPP + EMD (*n* = 24)	CAL gain at 12 months: 4.09 ± 1.68 mm (Group A) vs. 3.79 ± 1.67 (Group B)	The results have shown no significant differences in clinical outcomes when using EMD, regardless of the surgical technique employed.

**Table 5:** Evidence supporting the use of lasers in periodontal therapy. Abbreviations: 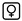
—women, 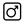;—men, SRP—scaling and root planing, AB—antibiotics (systemic administration), GCF—gingival crevicular fluid, MMP—matrix metalloproteinase, LANAP—Laser-Assisted New Attachment Procedure, LLLT—Low-Level Laser Therapy, ICG—indocyanine green, aPDT—antimicrobial photodynamic therapy, FMUD—full-mouth ultrasonic debridement, mSBI—modified sulcus bleeding index, NT—no treatment.

Study	No. of Patients	Group A	Group B	Group C	Outcome	Conclusion
Anna, S. et al. [77]	36 (24 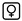 and 12 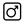 )	SRP + AB (*n* = 18)	SRP + PDT (*n* = 18)	-	MMP-8 levels at 6 months: 13.23 ± 8.71 (Group A) vs. 30.32 ± 29.77 (Group B)	Adjunctive systemic administration of amoxicillin and metronidazole is more effective in reducing GCF MMP-8 levels compared to the adjunctive use of PDT
Fadime, K.D. et al. [78]	60	SRP alone (*n* = 20)	LANAP (*n* = 20)	LLLT (*n* = 20)	CAL gain Median (IQR) at 3 months: 2 (−2/7) (Group A) vs. 3 (−3/8) (Group B) vs. 4 (−3/9) (Group C)	Laser-treated groups provide additional benefits to SRP. The application of LLLT positively affected recession.
Marco, A. et al. [82]	24 (15 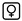 and 9 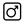 )	ICG-aPDT (*n* = 12)	off-mode aPDT (*n* = 12)	-	CAL gain at 6 months: 1.06 ± 1.63 mm (Group A) vs. 0.77 ± 0.81 mm (Group B)	Repeated ICG-aPDT combined with FMUD showed no additional overall benefit compared to FMUD alone, apart from some selective clinical and microbiological improvements.
Qiaoru, Z. et al. [79]	58, 70 implants	LLLT (*n* = 35)	NT (*n* = 35)	-	mSBI at 14 day: 0.63 ± 0.35 (Group A) vs. 0.84 ± 0.35 (Group B)	LLLT (Nd: YAG, 1064 nm) supports soft tissue healing after implantation, reduces postoperative pain, and enhances clinical outcomes.
Selma, D. et al. [80]	40 (18 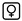 and 22 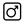 )	SRP + LLLT (*n* = 20)	SRP alone (*n* = 20)	-	CAL at 3 months: 3.18 ± 1.54 mm (Group A) vs. 3.15 ± 1.58 mm (Group B)	LLLT (Diode Laser 980 nm) with the chosen settings did not show a beneficial effect during the initial nonsurgical treatment of periodontitis.
Saurabh H, S. et al. [83]	60 sites	SRP + aPDT (*n* = 30)	SRP alone (*n* = 30)	-	PPD at 3 months: 2.23 ± 0.67 mm (Group A) vs. 3.67 ± 0.75 mm (Group B)	In patients with chronic periodontitis, clinical outcomes of conventional SRP can be improved by adjunctive PDT (using 810 nm diode laser and Indocyanine green as photosensitizer).
Suryakanth, M. et al. [84]	24 (9 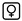 and 15 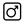 )	SRP only (*n* = 24)	SRP and aPDT (*n* = 24)	SRP, PDT, and LLLT (*n* = 24)	CAL gain at 6 months: 2.63 ± 0.47 (Group A) vs. 2.55 ± 0.44 (Group B) vs. 3.07 ± 0.55 (Group C)	A single application of PDT (with a 980 nm laser and methylene blue), combined with LLLT, offered added clinical benefits to SRP at 6 months post-treatment.
Urbashi, R.C. et al. [75]	24 (10 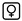 and 14 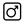 ), 130 teeth	SRP + aPDT (*n* = 66)	SRP alone (*n* = 64)	-	PPD at 3 months: 3.33 ± 0.31 (Group A) vs. 2.84 ± 0.44 (Group B)	ICG-based aPDT (810 nm diode laser) did not show additional advantage over SRP alone at 3 months.
Yikuan, W. et al. [85]	27 (14 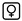 and 13 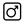 ), 74 sites	SRP + aPDT (*n* = 42)	SRP alone (*n* = 43)	-	PPD reduction at 3 months: 1.26 ± 0.62 (Group A) vs. 0.95 ± 0.65 (Group B)	Repeated PDT-assisted SRP improves soft tissue healing and comfort in Class II furcation

## Data Availability

The original contributions presented in this study are included in the article. Further inquiries can be directed to the corresponding author.

## Abbreviations

The following abbreviations are used in this manuscript:

aPDT	Antimicrobial Photodynamic Therapy
AB	Antibiotics (systemic administration)
AA	Ascorbic Acid
ABG	Autologous Bone Graft
ADM	Acellular Dermal Matrix
ATP	Adenosine Triphosphate
A-PRF	Advanced Platelet-Rich Fibrin
BMP	Bone Morphogenetic Protein
BM-MSCs	Bone Marrow-Derived Mesenchymal Stem Cells
CAL	Clinical Attachment Level
CAF	Coronally Advanced Flap
CCO	Cytochrome c Oxidase
CGF	Concentrated Growth Factor
CLN	Clindamycin
CM	Collagen Membrane
CTG	Connective Tissue Graft
CXP	Creos Xenoprotect
DBBM	Deproteinized Bovine Bone Mineral
DFDBA	Demineralized Freeze-Dried Bone Allograft
DPBM	Deproteinized Porcine Bone Material
DPSCs	Dental Pulp Stem Cells
ECM	Extracellular Matrix
e-PTFE	Expanded Polytetrafluoroethylene
EFP	European Federation of Periodontology
EMD	Enamel Matrix Derivative
FMUD	Full-Mouth Ultrasonic Debridement
FGG	Free Gingival Graft
GBR	Guided Bone Regeneration
GCF	Gingival Crevicular Fluid
GMSCs	Gingival Mesenchymal Stem Cells
GPCGF	Gel Phase Concentrated Growth Factor
GTR	Guided Tissue Regeneration
HA	Hyaluronic Acid
ICG	Indocyanine Green
ICG-aPDT	Indocyanine Green-Mediated Antimicrobial Photodynamic Therapy
I-PRF	Injectable Platelet-Rich Fibrin
LANAP	Laser-Assisted New Attachment Procedure
LLLT	Low-Level Laser Therapy
L-PRF	Leukocyte-Platelet-Rich Fibrin
LPCGF	Liquid Phase Concentrated Growth Factor
MAGR	Multiple Adjacent Gingival Recessions
MARF	Modified Apically Repositioned Flap
MIST	Minimally Invasive Surgical Technique
M-MIST	Modified Minimally Invasive Surgical Technique
MMP	Matrix Metalloproteinase
MPM	Modified Perforated Membrane
MSCs	Mesenchymal Stem Cells
MTZ	Metronidazole
MPP	Modified Papilla Preservation Technique
NT	No Treatment
OFD	Open-Flap Debridement
PB-MSCs	Peripheral Blood Mesenchymal Stem Cells
PC	Platelet Concentrates
PDGF	Platelet-Derived Growth Factor
PDPCs	Periosteum-Derived Progenitor Cells
PDL	Periodontal Ligament
PMPR	Professional Mechanical Plaque Removal
PRF	Platelet-Rich Fibrin
PRP	Platelet-Rich Plasma
PPD	Probing Pocket Depth
PTFE	Polytetrafluoroethylene
RBC	Red Blood Cell
RC	Recession Coverage
RDD	Radiographic Defect Depth
SBS	Synthetic Bone Substitute
SPP	Simplified Papilla Preservation Technique
SRP	Scaling and Root Planing
TGF-β	Transforming Growth Factor Beta
T-PRF	Titanium Platelet-Rich Fibrin
VBG	Vertical Bone Gain
WBC	White Blood Cell
xHyA	Cross-Linked Hyaluronic Acid
